# Editorial: Variation in Phase II Metabolism of Sex Steroids – Causes and Consequences

**DOI:** 10.3389/fendo.2015.00050

**Published:** 2015-04-28

**Authors:** Jenny Jakobsson Schulze, Lena Ekström

**Affiliations:** ^1^Karolinska Institutet, Stockholm, Sweden

**Keywords:** phase II, UGT, doping in sports, testosterone, cancer

Phase II metabolism, usually known as conjugation reactions, generate metabolites that are, in most cases, biologically inactive and subsequently excreted in bile or urine. Androgenic and estrogenic sex steroids are mainly inactivated by sulfation or glucuronidation by the enzyme families’ sulfotransferases (SULTs) or uridine diphospho glucuronosyltransferases (UGTs). Genetic variations in UGTs and SULTs have been implicated to play a role in hormone-dependent diseases and in the outcome for doping test results. Moreover, the use of drugs may interact with UGTs and SULTs and hence affect the phase II metabolism. The aim of this research topic forum was to highlight the progress made in this field via review papers and original articles as well as to promote future research with the aim to further understand the consequences of inter-individual difference in phase II metabolism and regulation of sex steroids.

Eight articles are included in this Research Topic, of which three are reviews and five are original research articles. In the original article written by Bang et al. (1), the UGT2B17 deletion polymorphism impact on testosterone replacement therapy was studied. There was no association with UGT2B17 genotype and testosterone serum peak levels, whereas the increase in testosterone from week 8 to 18 differed, subjects homozygous for deletion (del/del) had a smaller increase as compared to UGT2B17 carriers. Moreover, an association between LH levels and UGT2B17 genotype was found; del/del subjects exhibit lower levels of LH during treatment period. Testosterone is not only being therapeutically used but also a common drug together with synthetic androgens, i.e., nandrolone to abuse (doping). In the article by Strahm et al. (2), it was shown that a well characterized single nucleotide polymorphism in UGT2B15 is associated with the glucurunidation activity of 19-noradrosterone, the nandrolone metabolite analyzed at the WADA accredited doping labs. The significance of different phase II polymorphisms in female athletes was studied by Schulze et al. (3). They confirmed that UGT2B17 deletion polymorphism is an important determinant of T/E (a biomarker for testosterone doping) in women. Moreover, the use of hormonal contraceptives was shown to affect the T/E ratio, which will be important to consider in doping test programs. The fact that drug use can affect the phase II metabolism and consequently doping test results were also investigated in the original article by Lundmark et al. (4). They found that the use of NSAIDs (diclofenac and ibuprofen) did not interact with the urinary excretion of testosterone- and epitestosterone-glucuronides, and hence have no impact on T/E ratio. In the mini review by Jenkinson et al. (5), it was noted that dietary compounds such as white and green tea inhibit testosterone UGT2B17-derived glucuronidation activity *in vitro*. Hence, it is possible that in addition to drug use, the diet may influence the UGTs and alter the risk of hormone-related diseases and impacting doping test results; however, this needs to be verified *in vivo*.

Even though most androgens are excreted as glucuronides, some are also subject to sulfate conjugations. We have in Schulze et al. identified a new copy number variation polymorphism in the SULT2A1 gene (6). This CNV alters the capacity to excrete testosterone and some of its metabolites in the urine. Insertions are associated with higher excretion rate, both at basal level and after the administration of 500 mg testosterone enanthate. It is possible that this polymorphism may alter the risk of hormone-related diseases. In the mini review by McNamara et al., the latest findings in phase II metabolism in relation to breast and prostate cancer are discussed (7). Steroid sulfatase (STS), enzymes involved in the de-conjugation of sulfated compounds, was highlighted. In addition to phase II enzymes, steroid metabolizing phase I enzymes may determine the bioavailability of hormones. Mungenast et al. published an extensive review about the metabolism as well as the receptor effects of estrogens in relation to ovarian cancer, discussing strategies to target these pathways (8).

We believe that the articles included in this Research Topic have increased the knowledge on how genetic variation and drug use may impact phase II metabolism in general and doping test results in particular. Thanks to the open access policy of this journal, these articles will reach a broad audience and we hope it will encourage researcher to continue to conduct studies of phase II metabolism of hormones in relation to hormone-related disease, drugs, and doping test analysis.

## Conflict of Interest Statement

The authors declare that the research was conducted in the absence of any commercial or financial relationships that could be construed as a potential conflict of interest.
